# Carbon sequestration by Australian tidal marshes

**DOI:** 10.1038/srep44071

**Published:** 2017-03-10

**Authors:** Peter I. Macreadie, Q. R. Ollivier, J. J. Kelleway, O. Serrano, P. E. Carnell, C. J. Ewers Lewis, T. B. Atwood, J. Sanderman, J. Baldock, R. M. Connolly, C. M. Duarte, P. S. Lavery, A. Steven, C. E. Lovelock

**Affiliations:** 1 Deakin University, School of Life and Environmental Sciences, Centre for Integrative Ecology, 221 Burwood Highway, Burwood, VIC 3125, Australia; 2 Climate Change Cluster, University of Technology Sydney, 2007 Australia; 3 Department of Environmental Sciences, Macquarie University, Sydney, NSW 2109, Australia; 4 School of Science & Centre for Marine Ecosystems Research, Edith Cowan University, Joondalup, WA 6027, Australia; 5 UWA Oceans Institute, University of Western Australia, Crawley, WA, Australia; 6 Department of Watershed Sciences and The Ecology Center, Utah State University, Logan, UT 84322 USA; 7 Global Change Institute, University of Queensland, St Lucia, Queensland 4072, Australia; 8 Woods Hole Research Center, 149 Woods Hole Road, Falmouth MA 02540, USA; 9 CSIRO Agriculture and Food, PMB2, Glen Osmond, SA 5064, Australia; 10 Australian Rivers Institute – Coast & Estuaries, and School of Environment, Gold Coast campus, Griffith University, Queensland, 4222 Australia; 11 King Abdullah University of Science and Technology (KAUST), Red Sea Research Center (RSRC), Thuwal, 23955-6900, Saudi Arabia; 12 Centro de Estudios Avanzados de Blanes, Consejo Superior de Investigaciones Científicas, Blanes 17300, Spain; 13 CSIRO Ocean and Atmosphere Flagship, Ecosciences Precinct, Brisbane, QLD 4001, Australia; 14 The School of Biological Sciences, The University of Queensland, St Lucia QLD 4072.

## Abstract

Australia’s tidal marshes have suffered significant losses but their recently recognised importance in CO_2_ sequestration is creating opportunities for their protection and restoration. We compiled all available data on soil organic carbon (OC) storage in Australia’s tidal marshes (323 cores). OC stocks in the surface 1 m averaged 165.41 (SE 6.96) Mg OC ha^−1^ (range 14–963 Mg OC ha^−1^). The mean OC accumulation rate was 0.55 ± 0.02 Mg OC ha^−1^ yr^−1^. Geomorphology was the most important predictor of OC stocks, with fluvial sites having twice the stock of OC as seaward sites. Australia’s 1.4 million hectares of tidal marshes contain an estimated 212 million tonnes of OC in the surface 1 m, with a potential CO_2_-equivalent value of $USD7.19 billion. Annual sequestration is 0.75 Tg OC yr^−1^, with a CO_2_-equivalent value of $USD28.02 million per annum. This study provides the most comprehensive estimates of tidal marsh blue carbon in Australia, and illustrates their importance in climate change mitigation and adaptation, acting as CO_2_ sinks and buffering the impacts of rising sea level. We outline potential further development of carbon offset schemes to restore the sequestration capacity and other ecosystem services provided by Australia tidal marshes.

Removal of atmospheric carbon dioxide (CO_2_) through sequestration in natural ecosystems is necessary to keep global temperature under 2 °C of pre-industrial levels as the world transitions to a low-carbon economy. Whereas technological approaches to remove atmospheric CO_2_ are being sought, biosequestration of CO_2_ is already a proven process that can be conserved, enhanced and incorporated into emission accounting and reduction frameworks. Vegetated coastal habitats – tidal marshes, seagrasses and mangroves – known as ‘blue carbon ecosystems’, rank among the most efficient biosequestration systems on the planet^1^. During the climate change negotiation meetings in Paris at COP21, Australia followed other nations in announcing that it will “*increase understanding of, and accelerate action on the important role of coastal blue carbon ecosystems in climate change action*”. It will also be among the first countries to include blue carbon in its national greenhouse gas inventory. For these aspirations to be fulfilled, Australia needs to: 1) determine the stocks and distribution of blue carbon; 2) determine the factors controlling the accumulation of blue carbon around its coastline; and 3) identify and capitalise on opportunities for implementing management actions capable of enhancing carbon stocks or avoiding greenhouse gas emissions above the ‘business as usual’ scenario. Here we contribute to these goals, with a focus on tidal marshes (also referred to commonly as ‘saltmarshes’).

Australia has vast tracts of tidal marshes, with a total area estimated at 13,825 km^2^ (1,382,500 hectares). Australian tidal marshes have long been considered a poorly understood and neglected resource^2^. Since European settlement in Australia (1788), vast areas of tidal marsh have been cleared for agricultural and urban development (Table 1). Tidal marshes are regarded as one of the 10 major terrestrial and marine ecosystems in Australia most vulnerable to exhibiting tipping points, where relatively small changes in the environment lead to disproportionately large ecosystem losses^3^. Protection and conservation of Australian tidal marsh is now improving^4^. For example, through multiple legislative acts Australian coastal marshes have been declared either endangered or vulnerable ecological communities^2^, affording them a higher level of protection and conservation than non-threatened ecosystems. Still, many areas have been lost or remain denuded, making rehabilitation and restoration of Australia’s tidal marshes a task of primary importance.

An opportunity now exists to finance the restoration of tidal marshes through a growing carbon-offset market focused around nature-based climate mitigation (via biosequestration)^5^. Indeed, a recent paper by Rogers *et al*.^6^ proposed a series of actions that could improve protection of tidal wetlands and their ecosystem services by building interest in the recently-recognised value of tidal marshes in carbon offset markets. Tidal marshes are among the Earth’s most efficient carbon sinks. They accumulate organic carbon in their soils at rates up to 55-times faster than tropical rainforests, and store the carbon in soils for millennial timescales^1^. However, human activities can diminish the capacity of tidal marshes to sequester blue carbon^7^, and loss of blue carbon from tidal marsh ecosystems has been reported due to land reclamation^8^, chemical and physical disturbances^9^,^10^, eutrophication^11^, and loss of top-down control by the removal of associated fauna^12^,^13^. Sea level rise and climate change may also influence carbon sequestration within tidal marshes either positively or negatively^14^,^15^.

Information on Australian tidal marsh carbon stocks and accumulation rates has increased considerably over the past decade through localised efforts. Carbon stocks and accumulation rates for Australian tidal marshes appear to be comparable with global average values^16^,^17^,^18^,^19^,^20^, although previous Australian data have been limited in both geographical distribution and sample size. Carbon stocks and accumulation rates within tidal marshes vary with plant community types and their geomorphic environment due to differences in their abilities to trap and retain allochthonous carbon from fluvial inputs and tidal transport^17^,^20^. For this reason, human modifications to catchments that affect either fluvial inputs (e.g. clearing or dams) or tidal exchange (artificial levees) can significantly affect OC sequestration rates and tidal marsh resilience^4^,^17^.

The primary goal of this study was to provide new estimates of blue carbon stocks and accumulation rates for Australian tidal marshes. A large new (previously unpublished) dataset comprising of sediment cores taken from the temperate coasts of Australia has been complied, along with all available literature data (Fig. 1). This represents by far the largest compilation of Australian tidal marsh OC data to date. In addition to quantifying OC stocks and accumulation rates, we investigated possible factors (i.e. climate and habitat characteristics) driving carbon storage in tidal marshes. The work focused on OC located below-ground because much of the above-ground plant material is not sequestered in the long-term, and, most importantly, because the highest proportion of the carbon stock is in the sedimentary, below-ground pool^21^.

## Results

A total of 323 samples were analysed from the states of Victoria (VIC, 45 locations), New South Wales (NSW, 25 locations), temperate Queensland (QLD, 6 locations), Western Australia (WA, 4 locations), South Australia (SA, 3 locations) and Northern Territory (NT, 1 location). A total of 11 observations of tidal marsh carbon stocks were sourced from the literature. All data are summarised in Appendix 1. Estuary type included barrier estuaries, saline coastal lagoons, drowned river valleys/estuaries, sand islands, sand flats, tidal creeks, embayments, riverine estuaries, marine inlets, estuarine lakes, and periodically inundated lakes. The dominant vegetation types were: *Sarcocornia quinqueflora* (117 cores), *Juncus kraussii* (84 cores), *Sporobolus virginicus* (40 cores), *Suaeda australis* (14 cores), *Tecticornia sp.* (13 cores), *Baumea juncea* (6 cores) and *Paspalum vaginatum* (3 cores). Another 43 cores had a mix of species, and 3 cores did not have vegetation data.

Carbon stocks varied from 14 to 962 Mg OC ha^−1^ in the top 1 m, with a mean of 165.41 Mg OC ha^−1^ (SE 6.96). Carbon stocks at 30 cm depth varied from 8.89 to 603.67 Mg OC ha^−1^, with a mean of 77.92 Mg OC ha^−1^ (SE 3.35). Australian tidal marsh sediment vertical accretion rates ranged from 0.33 to 5.93 mm yr^−1^, with an overall mean of 2.09 ± 0.32 mm yr^−1^, while surface elevation tables (SET’s) recorded a mean elevation change of 1.44 mm yr^−1^ (SE 0.44, Appendix 2). We calculated a mean Australian tidal marsh carbon accumulation rate of 54.52 g OC m^2^ yr^−1^ (SE 2.34; 0.55 Mg OC ha^−1^ yr^−1^) (see methods, Equation 1). We therefore calculated that Australian tidal marshes sequester carbon in their sediments at a rate of 0.75 Tg C yr^−1^, or 750,477 tonnes C yr^−1^, which has a value of $AUD33.33 million ($USD28.02 million as September 2016) at a weighted average price of $AUD12.10 per Mg CO_2_-e (calculated over the last three auctions held by Australia’s Clean Energy Regulator).

On a state-by-state basis, we found New South Wales tidal marshes to have the lowest mean percent OC (5.35 ± 0.49%, Table 2) but the highest mean dry bulk density (0.92 ± 0.03 g cm^3^) and carbon stock (188.3 ± 9.92 Mg OC ha^−1^, 1 m depth, Table 2). Queensland had the highest mean percent OC (8.67 ± 2.17, Table 2), while Western Australia had the lowest mean OC stocks (91.25 ± 10.24 Mg OC ha^−1^, 1 m depth, Table 2). NSW had higher mean OC stocks on a per area basis than VIC (Table 2), although VIC had more OC overall due to a larger total area of tidal marsh (Fig. 2). Queensland had the highest estimated total OC stock in the top 1 m at 99,126 Gg, although this estimate had large variation around the mean value (Fig. 2).

Geomorphic setting was found to be a strong predictor of OC stocks with fluvially-influenced tidal marsh sites having approximately double the OC than of sites with greater marine influence (ANOVA, *F*_(1,186)_ = 84.262, *P* = 0.000, Fig. 2). This pattern was consistent across the states that were analysed; NSW and VIC (ANOVA, *F*_(1,186)_ = 2.094, *P* = 0.15, Fig. 3). While organic carbon stocks did not differ at sites dominated by *Juncus kraussii, Sarcocornia quenqueflora* and mixed vegetation (69.59 ± 3.89, 80.25 ± 4.95, 83.9 ± 3.96 Mg OC ha^−1^ respectively), *Sporobolus virginicus* dominated sites were found to be significantly lower (61.48 ± 7.46 Mg OC ha^−1^, lme, *t*_(199)_ = −2.757, *P* = 0.006). A linear mixed-effects model detected no significant relationship between mean maximum temperature (°C) and carbon stocks (*t*_(243)_ = −1.515, *P* = 0.13).

Australian tidal marshes were found to contain an estimated 210.98 Mg (million tonnes) of OC in the top 1 m, based on an area of 1,376,500 ha (see Methods for calculation).

## Discussion

In this study we collected data on OC stocks from 84 tidal marsh sites (323 soil cores) around Australia – the most comprehensive Australian tidal marsh OC database to date – providing a two fold increase in estimates since global data published by Chmura *et al*.^22^ and Ouyang and Lee^18^. Previous studies on OC stocks for Australian tidal marshes have only compared across multiple estuaries^17^,^19^, but here we significantly expand this spatial coverage to a regional, and national scale. Only one previous study, by Ouyang and Lee^18^, has estimated Australia-wide tidal marsh sequestration and was derived from just 3 observations in NSW and VIC. Ouyang and Lee^18^ estimated that Australian tidal marshes sequester 274.8 g C m^−2^ yr^−1^, equating to 3.78 Tg C yr^−1^ based on a tidal marsh area of 13,765 km^2^ (1,376,500 ha). Our estimates are lower than this; we find that Australian tidal marshes sequester 54.52 g OC m^2^ yr^−1^, which equates to 0.75 Tg OC yr^−1^.

We note, however, that the two studies used by Ouyang and Lee^18^ to calculate the rate of tidal marsh carbon accumulation appear to have led to a 2.6-fold overestimate of the carbon sequestration by Australian tidal marshes. Ouyang and Lee^18^ included in their estimate a value from Howe *et al*.^23^ for highly disturbed tidal marshes, which had a 2.14 times higher C sequestration rate than their undisturbed site, and with only 3 observations included in the mean it is possible this led to an overall overestimation. In addition, Ouyang and Lee^18^ omitted Saintilan’s low carbon accumulation estimate for *Juncus* tidal marsh, although they included it in their summary table. After accounting for these corrections, Ouyang and Lee’s^18^ estimate of carbon accumulation by Australian marshes would be lower, at 105.7 g C m^−2^ yr^−1^. Nevertheless, our estimates remain below current global means for tidal marsh OC sequestration reported by Ouyang and Lee (244.7 g C m^−2^ yr^−1^, n = 143)^18^, Duarte *et al*. (151 g C m^−2^ yr^−1^, n = 96)^24^ and Chmura *et al*. (210 g C m^−2^ yr^−1^, n = 116 – included mangroves)^22^. That said, most of the world’s tidal marshes remain to be sampled.

Compared with Ouyang and Lee^18^, our values for %OC and dry bulk density (g cm^3^, DBD) were generally in the same range and, therefore, we suggest that variance in sediment accretion (mm yr^−1^) between previous global estimates is the likely driver of the comparatively low OC sequestration rates observed here. In this study the mean sediment accretion rate was 2.09 mm yr^−1^ which was based on 20 observations (Appendix 2), whereas Ouyang and Lee^18^ used literature values of 3.4 mm yr^−1^, 9.8 mm yr^−1^ from Howe *et al*.^25^, and 1.8 mm yr^−1^ from Saintilan *et al*.^17^ (3 observations in total), resulting in a comparatively higher OC accumulation estimate (5 mm yr^−1^). Likewise, Duarte *et al*.^26^ reported a mean ± SE sediment accretion rate for 98 tidal marshes around the world to be 6.73 ± 0.07 mm yr^−1^, three-fold higher than the mean value we report for Australian marshes. Much of this data was from North America and Europe where tidal marshes are dominated by *Spartina alterniflora*, which occurs lower in the mid-intertidal zone, approximately at mean sea level^27^. Although elevation data was not collected across our sampling locations, mainland Australian marshes generally occur relatively higher in the intertidal or supratidal zone, often at the landward edge of mangrove forests^14^,^28^,^29^,^30^. As sediment delivery is higher at locations lower in the intertidal zone^31^ differences in sediment accretion between Australian regions with those dominated by *Spartina sp.* may be associated with differences in the position of tidal marshes in the intertidal. Elevation differences could also be limiting our comparison of carbon stocks among Australian marsh species, with *Juncus* often (though not always) occurring at higher elevations within the same marsh than *Sarcocornia.* Obtaining more data on the position of Australian saltmarshes within the tidal frame and assessing the impact of this upon sediment accumulation rates and ecogeomorphic responses is clearly a research priority for reducing uncertainty in carbon accumulation rates for the region. The collection of data from severely under-represented large, macrotidal catchments (i.e. northern Australia) will also present an opportunity to investigate the role of tidal range and catchment characteristics on surface and carbon accumulation in Australian marshes.

The significantly lower OC stocks found beneath tidal marsh vegetation species *Sporobolus virginicus* (sand couch - perennial grass) indicate that like seagrasses, plant community composition in tidal marshes may be a major driver of sediment carbon concentrations^32^ Previous studies have attributed changes in sedimentary OC concentrations and longevity in blue carbon habitats to plant morphological differences (i.e leaf and root structure)^33^, elevation and tidal inundation^34^, sediment grain size beneath plant communities^35^ and proportion of recalcitrant tissues^36^. As areas dominated by mixed vegetation, *Sarconornia* and *Juncus sp.* were found to have similarly high OC stocks, and are found in both the low and high tidal zones, respectively, and *Sporobolus virginicus* with comparatively lower OC stocks is situated in the mid tidal zone, we can infer that tidal inundation was not the driver of OC stock variation in this study. We therefore recommend that further research into the effect of tidal marsh vegetation on soil OC focus on morphological and recalcitrant tissue variations among plant community types^36^.

The significant relationship between geomorphology and OC stocks, with fluvial tidal marsh sites (landward) having twice the amount of OC as marine (seaward) sites subject to little or no fluvial influence, is consistent with findings by Saintilan *et al*.^17^ and Kelleway *et al*.^19^ at sites within the same region, and by Chmura *et al*.^37^ for eastern Canada. Kelleway *et al*.^19^ found that fluvially-influenced sites contained finer-grained sediments and higher contributions of refractory allochthonous OC than seaward sites. Data for grain sizes and contributions of allochthonous OC to the C stocks were not available. Future studies should focus on detailed catchment-level modelling of rainfall’s effects on regional fluvial systems, and the subsequent variance in terrigenous OC availability to tidal marshes in order to more accurately tease apart these relationships.

We estimate the potential value of the carbon stock locked in Australian tidal marshes (165.41 ± 6.96 Mg OC ha^−1^, 1 m depth) to be $AUD9.37 billion ($USD7.19 billion). Of course, this stock can only be monetized under scenarios where the stock is vulnerable and payments can be received for avoided emissions (e.g. REDD+). In addition, we found that Australian tidal marshes accumulate OC annually at a rate of 54.52 ± 2.34 g OC m^2^ yr^−1^, which represents a financial value of $AUD33.32 ± 1.57 million ($USD28.02 million). Together, these values highlight the significant role Australian tidal marshes have played in biosequestration historically (i.e. the long-term development of the current OC stocks) and may continue to play into the future (i.e. through annual OC accumulation). Conversely, the size of these estimates also gives some indication of: 1) the magnitude of OC which may have been lost through past habitat destruction - with up to 70% of the pre-European extent of coastal wetlands lost in parts of eastern Australia^38^,^39^; and 2) the OC pool which may be at risk to future emission if surviving marshes are lost or degraded.

Sea level rise is likely to have particular implications for the distribution of Australian tidal marshes, and may influence OC stocks and the capacity of marshes to accumulate OC in the future. Our assessment has indicated that Australian tidal marshes accumulate sediments at a rate of 2.09 ± 0.32 mm yr^−1^, which is almost three times slower than marshes globally (6.73 ± 0.07 mm yr^−1^)^40^. While belowground processes (such as subsidence, root production/decomposition and groundwater dynamics) also influence ecosystem response to sea level rise, a predicted global rise in excess of 0.26 m (and up to 0.82 m) over the subsidence of the 21st century^41^ is likely to outpace marsh elevation building in at least some locations. Of course, the question of whether tidal marshes will keep pace with sea level rise cannot be predicted by comparing rates of contemporary accretion against rates of sea level rise – because, for example, subsidence may accelerate or allochthonous tidal marsh inputs may increase with accelerated sea level rise. If the subsequent response to sea level rise involves an ecosystem transition from tidal marsh to mangrove then this may lead to an increase in OC storage (as demonstrated recently in the Sydney region^15^), though broader investigation of this phenomenon is needed. Furthermore, little is known of the likely long-term fate of OC under drowning (i.e. without transition to mangrove).

Where there is suitable topography to enable upslope migration of marsh (including through the removal of anthropogenic barriers such as levees and floodgates), new areas of tidal marsh establishment may offer an opportunity to increase OC accumulation and storage under rising sea levels. Moreover, this value compounds with that derived from other ecosystem services of tidal marshes, such as their nursery role for many marine and bird species, and for their value in coastal protection and nutrient cycling. Indeed, the loss of tidal marshes around Australia (Table 1) also offers an opportunity to increase the value of tidal marshes by restoring some of the lost areas. Both conservation and restoration actions (e.g. reinstating tidal flow) should be considered to derive the full value of tidal marsh conservation as a sound strategy to derive climate change mitigation and adaptation co-benefits while conserving and restoring ecosystem values.

### Conclusions, caveats and recommendations

Australia is home to approximately 33% of the planet’s tidal marsh (based on an Australia area of 13,765 km^2^ and a global area of 41,657 km^2^ 18), however there has been widespread decline in tidal marsh distribution since European settlement (conversion for agriculture and coastal development). The addition of climate change (particularly through sea level rise and the associated phenomena of mangrove encroachment and ‘coastal squeeze’)^42^,^43^,^44^ represents a further threat to these ecosystems. Declines in tidal marsh area and condition is gaining increased attention thanks to recent appreciation of its valuable role in sequestering carbon, which is important to offsetting anthropogenic CO_2_ emissions. Australia’s recent decision to incorporate wetlands into the National Greenhouse Gas Inventory (announced at COP21, Paris) signifies an important step towards accounting for CO_2_ emissions and reductions in their carbon sequestration capacity associated with losses of tidal marsh and also for identifying opportunities for restoration of OC sequestration. This study represents a major advance in our understanding of carbon sequestration by Australian tidal marshes, with data from 323 sites around Australia, 45% of which are new data not published thus far. We stress that a large proportion of our OC stock data came from 30 cm cores, whereas international methodologies are generally based on 1 m depths (e.g. IPCC^45^ and blue carbon initiative^46^); we therefore extrapolated 30 cm cores to 1 m, necessary to allow comparison with published reports. We did our best to account for potential changes in OC stocks with depth in the 30–100 cm range (see Equations 3 and 4 in methods), but note that the extrapolation process could under- or over-estimate OC stocks.

**Figure 1:**
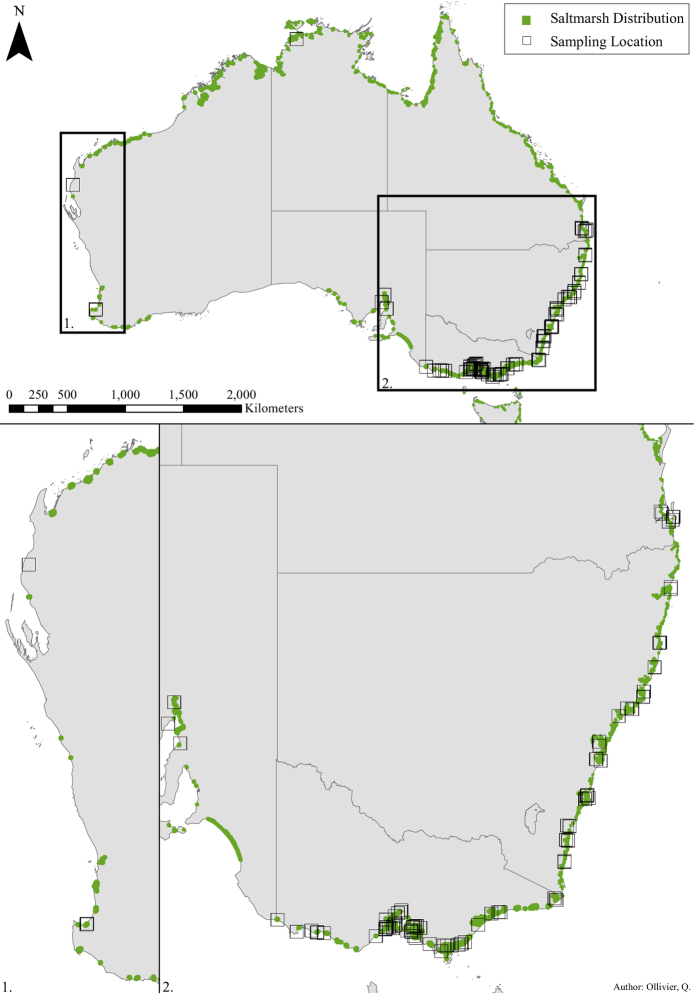
Sampling locations used to obtain data on organic carbon and other soil properties within temperate Australian tidal marshes. Total distribution of Australian tidal marshes is also displayed^56^,^57^,^58^,^59^,^60^,^61^,^62^,^63^,^64^. Figure created with ArcMap Version 10.2.2.

**Figure 2:**
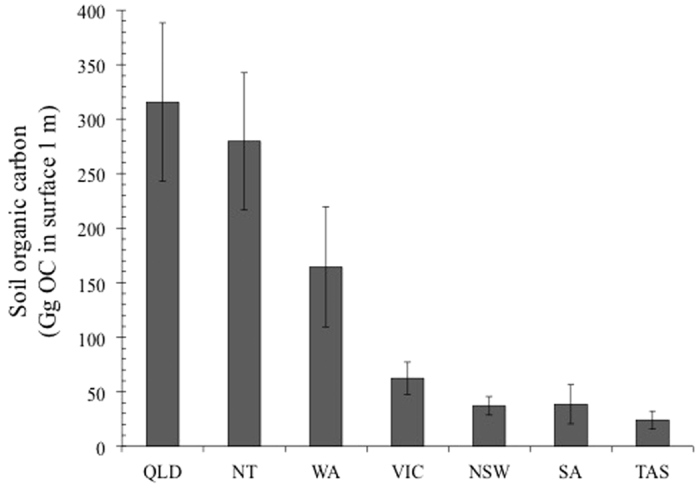
Estimated total organic carbon stored in Australian tidal marsh expressed in Gigagrams (Gg OC, mean ± standard error). State-specific tidal marsh areas were obtained from the literature (Table 1). Values shown here have been square root transformed for optimal visual comparisons. Due to a lack of carbon stock data in the Northern Territory and Tasmania, the mean of all other states combined were applied to their state-specific tidal marsh areas.

**Figure 3:**
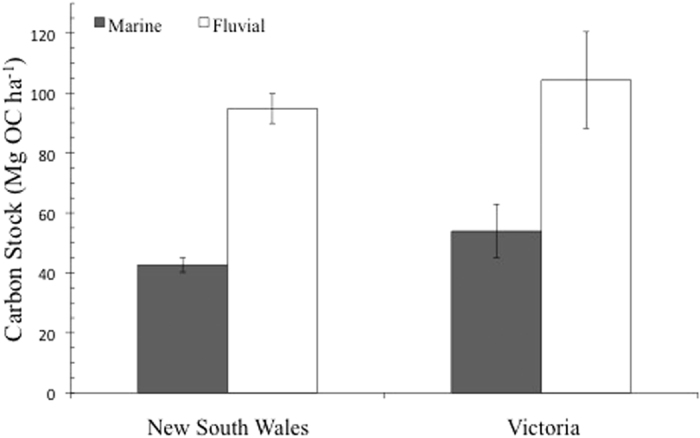
Tidal marsh carbon stocks (to 1 m, mean ± standard error) at sites with clear fluvial or marine influences. New South Wales: marine *n* = 77, fluvial *n* = 77. Victoria: marine *n* = 18, fluvial *n* = 18. Locations of comparisons and further information are shown in Appendix 1.

For future research we recommend the following:

- Collect data on OC stocks and accumulation rates for areas still under-represented in this study, including across northern Australia, Tasmania, South Australia and Western Australia (see Fig. 1).
- Develop and calibrate predictive models to predict the distribution of tidal marsh carbon, and how management actions will affect the persistence and carbon sequestration capacity of tidal marshes in the future under different scenarios, such as restoration and sea level rise^47^,^48^. Here we recommend an integrated approach of ecosystem-based management that recognises the connectivity between coastal and upland processes. Tidal marsh restoration is typically pursued to maximise a single ecosystem service (e.g. intercepting polluted runoff water, defense against sea level rise), and although secondary benefits often ensue, an integrated management approach can maximise a range of ecosystem services and achieve better overall outcomes^48^.
- Investigate carbon offset opportunities, including through the removal of anthropogenic structures that limit expansion, hydrological recovery of tidal exchange with tidal marshes and their retreat potential as sea levels rise^49^.
- Research into the effect of morphological and recalcitrant tissue variation of different tidal marsh vegetation types on soil OC sequestration rate and storage longevity^36^.
- Catchment-level modelling of rainfall effects on the carbon sequestration rate of fluvially-influenced tidal marshes.
- Quantify OC stocks of tidal marsh cores over depths of 1 m to be consistent methodologies of the IPCC^45^ and recent blue carbon initiatives^46^ and avoid the need to extrapolate estimates from surface cores.
- Determine the importance of biosequestration relative to other ecosystem services provided by tidal marshes (e.g. nutrient cycling, shoreline stabilisation, pollution control, biodiversity enhancement)^50^, and explore and develop markets that allow payments for a range of ecosystem services in addition to carbon.

As the world tries to transition to a low-carbon economy, there is growing opportunity to finance the conservation and protection of tidal marshes using the carbon offset market. The Verified Carbon Standard (VCS) Methodology for Tidal Wetland and Seagrass Restoration could attract carbon credits for tidal marsh restoration, but to our knowledge there have been no attempts by carbon offset providers to use the VCS scheme in Australia. At the national level, the Australian Government’s Emission Reduction Fund (ERF) is the primary policy relevant to carbon management. Considering the magnitude of OC storage and accumulation in Australian tidal marshes, we recommend their inclusion within this policy framework. This study demonstrates the value of tidal marshes as blue carbon sinks in Australia and the potential for further development of carbon offset schemes to restore the carbon sequestration capacity and other ecosystem services provided by these unique ecosystems.

## Methods

Data on OC and other sediment properties were compiled from the states of Western Australia (WA), South Australia (SA), Victoria (VIC), New South Wales (NSW), and temperate Queensland (QLD). The locations of sites are shown in Fig. 1. Data consisted of unpublished studies from the Marine and Coastal Carbon Biogeochemistry Cluster project (http://www.csiro.au/en/Research/OandA/Areas/Coastal-management/Coastal-Carbon-Cluster), with the exception of Queensland and NSW data which were derived from previously published work^15^,^19^,^28^. An overview of the data can be found in Appendix 1.

Sediment cores (ranging from 10 to 100 cm long) were sampled by means of percussion and rotation, or vibrocoring. The core barrels consisted of PVC or aluminium pipes (50 to 90 mm inside diameter) with sharpened ends to cut fibrous material and minimise compression during coring. All cores were sealed at both ends, transported to the laboratory and stored at 4 °C until processing. Cores in NSW were sampled at 0–20 cm, 20–50 cm, and 50–100 cm intervals, while cores in QLD were sampled at 0–30 cm, and in VIC soils were sampled at 0–2 cm, 14–16 cm, and 28–30 cm increments. WA and SA soils were sampled in increments ranging from 1 cm to 10 cm. Samples were weighed before and after oven drying to constant weight at 60 °C (DW) to determine bulk density. Soil increments were homogenised and ground into a fine powder using a ball mill. We focussed on bulk soil OC, which included belowground living plant biomass (e.g. roots and rhizomes).

The ‘Champagne test’^51^ was used to determine whether samples contained inorganic carbon. Subsequently, only those samples which contained sufficient inorganic carbon to cause bubbling were subject to acid washing (1 M HCl) to remove inorganic carbon. The gravimetric OC content of the sediment samples was measured via elemental analysers^52^ or predicted from mid-infrared (MIR) spectra acquired for the ground samples. For MIR predictions, a representative set of 200 samples was identified by applying the Kennard-Stone algorithm to a principal components analysis of the MIR spectra acquired for all samples. The gravimetric OC content of each of the 200 identified samples was measured using an elemental analyser. Partial least squares regression was used to derive an algorithm capable of predicting gravimetric OC contents from the acquired MIR spectra for all samples^53^.

Information on sediment accretion rates (mm yr^−1^) were obtained from previously published research using marker horizons^25^,^30^,^54^,^55^. Although useful, comparisons of OC accumulation rates (g C m^2^ yr^−1^) could not be statistically analysed in this study due to the use of an Australian tidal marsh mean sediment vertical accretion value (obtained from the literature) used universally in the below equation:


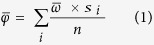


where 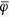 represents the average carbon accumulation rate found across Australian tidal marshes (g m^2^ yr^−1^), 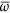 represents the average Australian tidal marsh sediment vertical accretion rate (mm yr^−1^) obtained from the literature (Appendix 2),s_*i*_ represents the conversion of individual core carbon stocks (Mg OC ha^−1^, 30 cm depth) to grams of OC per m^2^ in the top 1 mm soil layer, and *n* represents the number of individual cores. The value of *Australia’s total OC stock was calculated through the following equation:*


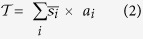


where 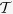 represents the total OC stored in Australian tidal marshes to 1 m depth, 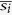 represents the mean OC stocks within each Australian state (Mg OC ha^−1^, 1 m depth), and α_*i*_ represents the total area of tidal marsh within each corresponding Australian state obtained from the literature (Table 1). This study did not sample within Tasmania, and only one site in the Northern Territory was found in the literature, so to calculate total stocks in these states the average OC stock values of all Australian locations were used as a proxy for 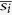 in the equation stated above.

To allow for direct comparisons with international literature, the OC stocks obtained in this study were standardized to 100 cm-thick using both logarithmic and linear regressions of OC concentrations with depth. Where samples were limited to 20 cm depth (due to difficulty penetrating deeper depths using available coring materials) extrapolation of regressions were only conducted to 30 cm (n = 5). Predictions of 1 m deep OC stocks were calculated as follows; Logarithmic regressions were applied to sediment cores with a depth between 50 < 100 cm allowing for both a higher *R*^2^, and core specific incorporation of carbon stabilisation effects often found beyond 30 cm depth. Linear regressions were applied to all cores of 30 cm depth, however, to account for likely carbon stabilization beyond 30 cm the following correction equation was applied (Eqs 3 and 4):


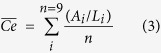



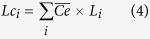


where 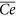 represents the mean of all core specific correction values, *A*_*i*_ represents the actual OC stock to 1 m depth of an individual core (Mg OC ha^−1^), *L*_*i*_ represents the linearly predicted OC stock value to 1 m depth of the same core (equation 3), or an individual core (equation 4) based on the surface 30 cm soils, *n* represents the number of available cores sampled to 1 m, and *Lc*_*i*_ represents the corrected 1 m stock value of an individual linearly predicted core. Port Augusta was not extrapolated to 1 m as the data obtained was only from the 0–10 cm soil layer.

To test the effects of geomorphic setting (fluvial or marine) only locations that retained clear delineation between geomorphic settings were used. For New South Wales (NSW), 77 paired comparisons of fluvial and marine settings within the same estuarine systems were used (e.g. upper and lower river sampling sites)^19^. In Victoria (VIC) not all sites were from the same estuary. To ensure consistency with NSW data, fluvial sites were chosen with similar distances from open-ocean (marine influence) (n = 18 fluvial, n = 18 marine, see Appendix 1. for the locations of VIC geomorphic comparisons). Due to large variation in dominant vegetation sample sizes, statistical analyses compared only the three most common species, *Juncus kraussii, Sarcocornia quinqueflora and Sporobolus virginicus*, and the ‘mixed’ category of sites with more than one dominating species. Normality was confirmed through visual examination of box plots and frequency histograms. A Levene’s Test confirmed homogeneity of variance between geomorphic settings (*P* > 0.05), however heterogeneous variance was found between dominant vegetation types (*P* < 0.05). A restricted maximum likelihood (REML) estimate of the parameters in a linear mixed-effects model (“lme()” function), with location included as a random factor, was used to assess the contribution of mean maximum temperature on tidal marsh OC stocks to a depth of 30 cm. A separate REML mixed effects model with the inclusion of fixed factor heterogeneity using the “varIdent()” function, again with location assessed as a random factor, was used to investigate the effects of dominant vegetation type on OC stocks to 30 cm depth. All assumptions of linear mixed-effects analysis were confirmed through regression diagnostics. All OC stock data were square root transformed, and two high outliers (Amity North and Amity South) were removed, to facilitate normality of OC stock values prior to analysis.

## Additional Information

**How to cite this article**: Macreadie, P. I. *et al*. Carbon sequestration by Australian tidal marshes. *Sci. Rep.* **7**, 44071; doi: 10.1038/srep44071 (2017).

**Publisher's note:** Springer Nature remains neutral with regard to jurisdictional claims in published maps and institutional affiliations.

## Supplementary Material

Supplementary Information File

## Figures and Tables

**Table 1:** Losses of Australian tidal marsh habitat by state according to available literature.

State/Territory	Area (km^2^)	Rate of loss (km^2^ yr^−1^)	Rate of loss ± SD (% total area yr^−1^)	Time period	Causes of Loss	Method	Reference(s)
**New South Wales**	73^65^	0.0931	0.01 ± 0.51%	1940s/1950s to 1990s	Incursion of terrestrial species, mangrove encroachment, reclamation.	Photogrammetric analysis of a subset of wetlands from nine estuaries within NSW	66
**Queensland**	5,322^67^	1.3510	0.0184%	Pre-1750 to 2011	Agriculture, urban & industrial development	Aerial imagery and ground-truthing of QLD coast	68,69
**South Australia**	84^67^	0.0824	4.4516%	1930s to 1970s & 1970s to 1990s	Mangrove encroachment, urban development	Photogrammetric analysis and ground-truthing for Gulf of St. Vincent, SA	70, 71, 72
**Tasmania**	37^67^	0.0406	0.2963%	1952 to 2006	Expansion of *Melaleuca ericifolia,* land clearing, levees (approx. 90%).	Aerial photo interpretation and ground-truthing of Circular Head, Tasmania (representing 20% of Tasmanian tidal marsh area)	73
**Victoria**	279^74^			Pre-1750 to 2008	Grazing, reclamation for agriculture, vehicle damage.	State-wide archival maps & field observations, aerial imagery & ground-truthing	74,75
Scenario I*		0.0423	0.0146% %				
Scenario II*		0.3334	0.0914				
**Western Australia**	2,965^67^	13.54	18%	1999–2002	Cyclone	Aerial imagery & photography, field validation	76
**Northern Territory**	5,005^67^	Unknown	Unknown	—	—	—	—

Literature that did not present values of the actual area lost was excluded from the summary. Total area lost per year in New South Wales is likely an underestimate, as this value is based on portions of selected estuaries only. There is no reliable information on tidal marsh loss for the Northern Territory. *Victoria state averages reflect two possible scenarios based on the ambiguity of tidal marsh in the Gippsland Lakes. “Scenario I: All ambiguous tidal marsh areas are natural. Scenario II: All the ambiguous tidal marsh areas are recent expansions, in which case they are counted as gains which offset other losses”^74^. The rate of tidal marsh loss for Western Australia presented here is based on an episodic event, and is therefore likely to be an overestimate of typical annual loss rates.

**Table 2:** Comparisons by state of organic carbon (OC) stocks and accretion rates across Australia (mean, SE).

State	Organic carbon (%OC)	Dry bulk density (g cm^3^)	Stock (Mg OC ha^−1^, 30 cm depth)	Stock (Mg OC ha^−1^, 1 m depth)	Sediment Accretion (g OC m^2^ yr^−1^)	Number of sites
NSW	5.35 ± 0.49	0.92 ± 0.03	69.5 ± 3.33	188.3 ± 9.92	48.63 ± 2.33	25
QLD	8.67 ± 2.17	0.73 ± 0.11	116.79 ± 8.72	186.26 ± 60.24	81.71 ± 26.43	6
SA	6.78 ± 1.56	0.24 ± 0.12	56.6 ± 8.7	169.78 ± 48.85	39.6 ± 6.1	3
VIC	7.86 ± 0.59	0.88 ± 0.04	86.86 ± 4.79	139.06 ± 7.88	60.77 ± 3.35	45
WA	7.35 ± 1.8	0.3 ± 0.09	43.12 ± 7.93	91.25 ± 10.24	30.16 ± 5.6	4

Dry bulk density and percent OC data is representative of 30 cm deep cores, except NSW where data is representative of 20 cm depth. Only OC stock values include data taken from the literature (Appendix 1). See methods for sediment accretion calculations, and Appendix 2 for accretion values.
