# Supplementary material for: Carbon sequestration by Australian tidal marshes
Source: Sci Rep. 2017 Mar 10;7:44071. doi: 10.1038/srep44071 (PMC5345033; doi:10.1038/srep44071)
Supplement: Supplementary Information File [file srep44071-s1.pdf]

Supplementary Information

Carbon sequestration by Australian tidal marshes

Peter I. Macreadie, Ollivier Q, Kelleway JJ, Serrano O, Carnell P, Ewers C, Atwood T, Sanderman J, Baldock J, Connolly RM, Duarte CM, Lavery P, Steven A, Lovelock C.E.

Appendix 1. Summary appendix. Organic carbon percent (%OC) and dry bulk density (DBD) within New South Wales are expressed as mean ± standard error of the 0 – 20 cm soil layer, all other states represent the 0 – 30 cm soil layer. See methods for 1 m deep carbon stock calculations.

Location	Estuary type	Geomorphic Setting	Dominant vegetation	Latitude	Longitude	Cores	Dry bulk density (g cm^{3})	Percent organic carbon (%OC)	Carbon Stock (Mg OC ha^{-1}, 30 cm depth)	Carbon Stock (Mg OC ha^{-1}, 1m depth)
**New South Wales**										
Berowra Creek[17]	Drowned valley estuary	-	*Juncus kraussii*	-33.626	151.12	1	-	-	84	309
Cararma Inlet[17]	Marine embayment	-	*Sarcocornia quinqueflora*	-34.985	150.772	1	-	-	64.5	124.5
Clarence River	Barrier estuary	Marine	*Sporobolus virginicus - Juncus kraussii*	-29.434	153.356	8	1.46 ± 0.03	0.75 ± 0.09	24.35 ± 2.42	36.83 ± 4.53
		Fluvial	*Sporobolus virginicus - Baumea juncea*	-29.379	153.352	8	1.26 ± 0.1	1.27 ± 0.29	49.98 ± 8.51	94.07 ± 16.24
Clyde River	Drowned valley estuary	Marine	*Sarcocornia quinqueflora - Juncus kraussii*	-35.710	150.172	8	1.22 ± 0.08	1.1 ± 0.32	34.87 ± 6.65	104.09 ± 20.93
		Fluvial	*Sarcocornia quinqueflora - Juncus kraussii -* Baumea juncea	-35.692	150.099	8	0.48 ± 0.08	11 ± 1.1	127.3 ± 7.72	420.18 ± 24.64
Crookhaven River	Barrier estuary	Marine	*Sporobolus virginicus - Juncus kraussii*	-34.909	150.741	8	1.26 ± 0.08	2.01 ± 0.55	56.84 ± 10.81	199.76 ± 21.18
		Fluvial	*Sporobolus virginicus - Juncus kraussii*	-34.935	150.714	8	0.66 ± 0.11	7.38 ± 1.05	110.53 ± 18.5	262.02 ± 42.3

Currambene Creek [17, 74]	Marine embayment – Riverine estuary	-	*Sarcocornia quinqueflora - Juncus kraussii*	-35.019	150.667	3	-	-	77.77 ± 29.62	139.35 ± 62.39
Georges River		Marine	*Sarcocornia quinqueflora - Sporobolus virginicus*	-34.01966	151.153076	3	0.99 ± 0.16	4.81 ± 2.18	39.59 ± 9.27	127.58 ± 29.88
		Fluvial	*Sarcocornia quinqueflora - Sporobolus virginicus*	-33.9847	151.010312	3	0.37 ± 0.9	9.05 ± 3.02	62.54 ± 11.95	201.52 ± 38.5
Kooragang Island [17]	Barrier estuary	-	*Sporobolus virginicus*	-32.848	151.72	1	-	-	97.5	161.5
Lake Cathie	Saline coastal lagoon	Marine	*Paspalum vaginatum - Juncus kraussii*	-31.549	152.850	8	1.21 ± 0.13	1.93 ± 0.64	41.24 ± 5.45	75.35 ± 8.97
		Fluvial	*Sarcocornia quinqueflora - Juncus kraussii*	-31.551	152.845	8	0.23 ± 0.03	20.43 ± 1.19	123.09 ± 9.31	340.36 ± 42.01
Macleay River	Barrier estuary	Marine	*Sporobolus virginicus - Juncus kraussii*	-30.882	153.021	8	0.96 ± 0.07	2.3 ± 0.22	58.29 ± 4.92	220.24 ± 16.61
		Fluvial	*Sporobolus virginicus - Juncus kraussii*	-30.894	152.999	8	1.09 ± 0.07	2.25 ± 0.4	56.24 ± 5.56	207.61 ± 14.08
Marramarra Creek [17]	Drowned valley estuary	-	*Juncus kraussii*	-33.523	151.1	1	-	-	93	335
Port Stephens	Drowned valley estuary	Marine	*Sporobolus virginicus - Sarcocornia quinqueflora*	-32.669	152.145	9	1.18 ± 0.07	1.63 ± 0.24	52.26 ± 4.77	181.68 ± 12.62

			- Juncus kraussi							
		Fluvial	*Sporobolus virginicus - Sarcocornia quinqueflora - Juncus kraussi*	-32.643	151.989	9	0.74 ± 0.08	3.13 ± 0.29	52.97 ± 6.49	220.08 ± 15.76
Tuross River	Barrier estuary	Marine	*Sarcocornia quinqueflora - Juncus kraussii*	-36.077	150.123	8	1.41 ± 0.05	0.64 ± 0.07	23.63 ± 2.25	69.33 ± 8.02
		Fluvial	*Sarcocornia quinqueflora - Juncus kraussii*	-36.055	150.063	8	0.39 ± 0.04	9.26 ± 0.52	96.79 ± 4.5	298.12 ± 22.63
Wallis Lake	Barrier estuary	Marine	*Sporobolus virginicus - Sarcocornia quinqueflora - Juncus kraussii*	-32.201	152.509	8	1.1 ± 0.09	2.43 ± 0.48	55.49 ± 9.34	102.08 ± 12.04
		Fluvial	*Sporobolus virginicus - Juncus kraussii*	-32.335	152.487	8	0.3 ± 0.03	17.8 ± 1.8	143.43 ± 5.57	422.42 ± 19.22
Wapengo Lake	Barrier estuary	Marine	*Sarcocornia quinqueflora - Juncus kraussii*	-36.620	150.015	9	1.21 ± 0.07	1.5 ± 0.34	38.03 ± 7.23	72.29 ± 14.94
		Fluvial	*Sarcocornia quinqueflora - Juncus kraussii*	-36.589	150.016	9	0.66 ± 0.05	8.99 ± 1.13	117.85 ± 8.32	215.51 ± 13.21
**Northern Territory**										
Middle Arm, Darwin Harbour [75]	Drowned valley estuary	-	Samphire - Saltflat	-12.6482	130.9639	1	-	-	46	69.77

**Queensland**										
Adams Beach	Sand island	-	*Juncus kraussii*	-27.52054	153.41237	3	0.14 ± 0.01	19.87 ± 0.26	46.48 ± 16.27	371.93 ± 296.01
Amity north	Sand island	-	*Juncus kraussii*	-27.43511	153.43516	3	0.45 ± 0.19	14.72 ± 7.19	221.07 ± 191.31	314.19 ± 212.88
Amity South	Sand island	-	*Juncus kraussii*	-27.43796	153.4342	3	0.41 ± 0.17	10.96 ± 6.01	181.77 ± 140.21	139.08 ± 34.42
Halloran Reserve	Tidal creek - embayment	-	*Sarcocornia quinqueflora*	-27.5699	153.29056	3	1.24 ± 0.04	0.81 ± 0.15	69.37 ± 21.4	64.43 ± 28.6
Nundah Creek	Tidal creek - embayment	-	*Sarcocornia quinqueflora*	-27.35169	153.08588	3	0.86 ± 0.16	4.07 ± 1.52	51.98 ± 17.99	87.6 ± 24.82
Tinchi Tamba Reserve	Tidal creek - embayment	-	*Sarcocornia quinqueflora*	-27.28858	153.04543	3	1.27 ± 0.05	1.60 ± 0.33	120.05 ± 52.38	140.35 ± 98.15
**South Australia**										
Port Augusta	Marine embayment - Riverine estuary	-	*Sarcocornia quinqueflora*	-32.47853	137.76463	1	0.52 ± 0.09	5.11 ± 1.11	62.73	N/A
Port Broughton	Marine inlet	-	*Sarcocornia quinqueflora*	-33.56679	137.949759	3	0.42 ± 0.17	6.86 ± 2.71	48.54 ± 12.65	146.89 ± 61.02
Whyalla	Marine embayment	-	*Juncus kraussii*	-33.04877	137.56947	1	0.34 ± 0.02	8.21 ± 0.22	74.66	238.46
**Victoria**										
Anderson Inlet	Riverine estuary - Marine inlet	-	*Sarcocornia quinqueflora*	-38.66	145.7896	3	0.97 ± 0.23	4.27 ± 2.33	64.04 ± 3.42	146.89 ± 61.02
Barwon River	Estuarine	-	*Tecticornia -*	-38.2476	144.499016	3	0.34 ±	13.11 ± 1.11	74.45 ± 175.56	116.66 ± 3.62

Estuary	lagoon - River estuary		*Sarcocornia quinqueflora*		7		0.05			
Cheetham Saltworks	Marine embayment	-	*Tecticornia - Sarcocornia quinqueflora*	-37.897267	144.7841	3	0.31 ± 0.05	13.73 ± 1.88	81.7 ± 10.32	129.96 ± 17.09
China Bay	Marine embayment	-	*Sarcocornia quinqueflora - Suaeda australis*	-38.22775	145.31115	3	0.22 ± 0.03	24.61 ± 4.13	124.45 ± 11.58	184.11 ± 29.64
Corner Inlet (1)	Marine embayment		*Tecticornia - Suaeda australis*	-38.689367	146.33515	3	1.36 ± 0.22	0.96 ± 0.39	TO DO	37.28 ± 4.77
Corner Inlet (2)	Marine embayment	Fluvial	*Tecticornia - Suaeda australis*	-38.69365	146.2436	3	0.77 ± 0.2	3.38 ± 1.59	TO DO	60.44 ± 20.13
Eagle Point Bay	River estuary - Barrier lagoon	-	*Sarcocornia quinqueflora*	-37.902534	147.71895	3	0.14 ± 0.02	37.72 ± 1.37	243.73 ± 25.67	382.55 ± 44.11
Fawthrop Lagoon (Wattle Hill Creek) Estuary	Coastal lagoon	-	*Sarcocornia quinqueflora - Suaeda australis*	-38.3541	141.60165	3	0.3 ± 0.03	13.1 ± 1.59	48.09 ± 5.38	73.71 ± 8.17
Glenelg Estuary - Oxbow Lake	Estuarine lake - Open	-	*Sarcocornia quinqueflora*	-38.06	141.001784	3	0.21 ± 0.03	18.99 ± 0.69	62.13 ± 6.36	94.87 ± 9.57
Jacks Beach[16]	Marine embayment	-	*Sarcocornia quinqueflora*	-38.340157	145.258526	1	-	-	79	173
	Estuarine	Fluvial		-38.182734	147.306484	3	0.42 ±	19.22 ± 1.05	210.78 ± 47.84	336.12 ± 77.62

Lake Coleman	lake - Open		*Tecticornia - Suaeda australis*				0.1			
Lake Connewarre Reserve (1)	Riverine Estuary	-	*Sarcocornia quinqueflora*	-38.250934	144.438117	3	0.52 ± 0.14	12.85 ± 5.51	58.31 ± 14.49	90.34 ± 24.33
Lake Connewarre Reserve (2)	Riverine estuary	Fluvial	*Sarcocornia quinqueflora*	-38.241234	144.4209	3	0.18 ± 0.02	25.92 ± 1.27	59.97 ± 4.71	93.27 ± 7.3
Lake King - Cunninghame Arm	Coastal lagoon	Marine	*Sarcocornia quinqueflora*	-37.875834	148.0101	3	0.77 ± 0.22	2.57 ± 1.25	26.36 ± 6.68	41.79 ± 10.37
Lakes Entrance - Maringa Creek	Creek estuary - Coastal lagoon	-	*Sarcocornia quinqueflora*	-37.870617	147.939184	3	0.15 ± 0.01	24.54 ± 1.99	128.63 ± 10.62	201.73 ± 17.18
Limeburners Lagoon	Riverine estuary	-	*Tecticornia - Sarcocornia quinqueflora*	-38.052767	144.411	3	0.7 ± 0.2	3.16 ± 0.9	106.26 ± 57.76	173.5 ± 98.1
Mallacoota Inlet - Bottom Lake	Marine inlet	Marine	*Tecticornia*	-37.566917	149.760817	3	1.5 ± 0.29	0.55 ± 0.07	18.92 ± 2.93	29.93 ± 4.74
Mallacoota Inlet - Top Lake	Marine inlet - Riverine estuary	Fluvial	*Sarcocornia quinqueflora*	-37.525967	149.69725	3	0.2 ± 0	20.51 ± 0.91	90.12 ± 5.98	143.18 ± 9.97
Merri River Estuary	Riverine estuary	-	*Sarcocornia quinqueflora*	-38.395034	142.467617	3	0.15 ± 0.01	33.4 ± 2.71	95.12 ± 8.89	148.59 ± 14.46
Moyne River	Riverine estuary	-	*Sarcocornia quinqueflora*	-38.3652	142.250617	3	0.26 ± 0.05	17.09 ± 3.71	96.21 ± 9.19	152.25 ± 14.33

Estuary - Belfast Lough (1)										
Moyne River Estuary - Belfast Lough (2)	Riverine estuary	Marine	*Suaeda australis*	-38.38045	142.239717	3	0.24 ± 0.04	16.35 ± 3.93	68.75 ± 9.34	106.93 ± 15.76
Nooramunga Marine and Coastal Park (1)	Marine inlet - Sand flats	-	*Sarcocornia quinqueflora*	-38.6752	146.691684	3	0.86 ± 0.07	2.95 ± 0.17	39.56 ± 3.66	61.46 ± 5.76
Nooramunga Marine and Coastal Park (2)	Marine inlet - Sand flats	-	*Sarcocornia quinqueflora*	-38.698817	146.46855	3	1.22 ± 0.24	0.7 ± 0.2	16.01 ± 3.57	24.94 ± 5.51
Nooramunga Marine and Coastal Park (3)	Marine Inlet - Sand flats	-	*Tecticornia - Sarcocornia quinqueflora*	-38.60935	146.88875	3	0.46 ± 0.15	17.1 ± 5.92	110.73 ± 22.11	175.42 ± 37.05
Nooramunga Marine and Coastal Park (4)	Marine inlet - Sand flats	-	*Sarcocornia quinqueflora*	-38.64635	146.780917	3	1.54 ± 0.03	0.58 ± 0.08	22.81 ± 3.68	36.08 ± 6.13
Painkalac Creek Estuary	Riverine estuary	-	*Tecticornia - Sarcocornia quinqueflora*	-38.466617	144.096634	3	0.29 ± 0.09	28.62 ± 6.08	113.61 ± 19.78	177.25 ± 31.34
Phillip Island	Marine embayment	Marine	*Sarcocornia quinqueflora*	-38.51	145.345117	3	0.48 ± 0.1	6.77 ± 1.55	73.85 ± 5.84	116.1 ± 9.15
Port Phillip Bay	Marine embayment	-	*Sarcocornia quinqueflora - Suaeda*	-37.8663	144.878984	3	0.19 ± 0.01	24.22 ± 2.04	98.06 ± 24.93	154.65 ± 39.66

			australis							
Rhyll Inlet	Marine embayment	-	*Sarcocornia quinqueflora*	-38.45945	145.2882	3	0.24 ± 0.03	25.32 ± 7.41	93.99 ± 9.29	213.61 ± 36.84
Salt Lagoon	Marine embayment	-	*Tecticornia - Sarcocornia quinqueflora*	-38.161167	144.7143	3	0.38 ± 0.07	14.79 ± 4	50.69 ± 4.7	77.26 ± 7.37
Shallow Inlet	Marine inlet	Marine	-	-38.836384	146.151417	3	1.18 ± 0.12	1.33 ± 0.5	31.93 ± 4.09	49.89 ± 6.16
Stony Point[16]	Marine embayment	-	*Sarcocornia quinqueflora*	-38.365613	145.213855	1	-	-	78.2	164.1
Swan Bay (1)	Marine embayment	-	*Sarcocornia quinqueflora*	-38.227717	144.651834	3	0.31 ± 0.02	13.83 ± 2.9	101.02 ± 12.5	157.34 ± 18.96
Swan Bay (2)	Marine embayment	-	*Tecticornia - Sarcocornia quinqueflora*	-38.2667	144.63605	3	0.27 ± 0.04	17.85 ± 5.89	71.78 ± 5.14	112.17 ± 8.82
Swan Bay (3)	Marine embayment	Marine	*Tecticornia - Suaeda australis*	-38.19715	144.698734	3	0.26 ± 0.07	4.84 ± 4.84	104.22 ± 31.89	177.42 ± 37.37
Thompson Creek Estuary (1)	Riverine estuary	-	*Sarcocornia quinqueflora - Suaeda australis*	-38.294967	144.386184	3	0.8 ± 0.03	4.7 ± 0.76	54.31 ± 2.27	83.77 ± 3.58
Thompson Creek Estuary (2)	Riverine estuary	Fluvial	*Sarcocornia quinqueflora*	-38.286484	144.400717	3	0.19 ± 0.02	33.01 ± 0.64	142.35 ± 27.41	223.12 ± 45.04
Tooradin Coastal Reserve	Marine embayment	Fluvial	*Sarcocornia quinqueflora - Suaeda*	-38.213267	145.376884	3	0.36 ± 0.16	13.36 ± 4.19	135.87 ± 23.38	133.76 ± 19.48

			australis							
Warringine Park	Marine embayment	-	*Tecticornia* - *Sarcocornia quinqueflora*	-38.3179	145.195717	3	0.22 ± 0	23.58 ± 2.78	114.61 ± 18.15	198.68 ± 19.07
Werribee River Estuary	Riverine estuary - Marine embayment	-	*Sarcocornia quinqueflora* - *Suaeda australis*	-37.970633	144.684283	3	0.6 ± 0.17	8.12 ± 1.33	57.63 ± 6.54	89.57 ± 10.39
Western Port Bay	Marine embayment	-	*Sarcocornia quinqueflora*	-38.260683	145.494967	2	0.91 ± 0.01	3.85 ± 0.17	88.66 ± 18.45	162.01 ± 8.37
Western Port Coastal Reserve	Marine embayment	-	*Suaeda australis*	-38.24955	145.248667	3	0.35 ± 0.09	13.03 ± 2.72	107.93 ± 6.38	170.26 ± 10.46
Yambuk Lake Estuary	ICO lake	-	*Sarcocornia quinqueflora*	-38.330133	142.064567	3	0.4 ± 0.12	10.65 ± 0.45	136.35 ± 8.08	217.84 ± 12.51
Yaringa National Park[16]	Marine embayment	-	*Sarcocornia quinqueflora*	-38.239763	145.258526	1	-	-	72.7	169.1
Yarra Estuary	Riverine estuary - Marine embayment	-	*Sarcocornia quinqueflora* - *Suaeda australis*	-37.8252	144.89635	3	0.46 ± 0.19	13.91 ± 2.55	172.28 ± 8.23	273.23 ± 12.33
**Western Australia**										
Lake Macleod	Periodically inundated lake	-	-	-23.95	113.633333	1	1.05 ± 0.07	0.74 ± 0.09	20.77	101.59
Wonnerup Inlet (1)	Marine lnlet	-	*Juncus kraussii*	-33.61362	115.42524	3	0.4 ± 0.09	11.08 ± 4.5	62.49 ± 24.15	92.23 ± 28.01

Wonnerup Inlet (2)	Marine inlet	-	*Juncus kraussii*	-33.62914	115.407914	3	0.68 ± 0.16	9.1 ± 2.47	37.51 ± 2.99	76.8 ± 16.06
Vasse Estuary	Riverine estuary	-	-	-33.61714	115.41707	3	0.94 ± 0.08	4.07 ± 0.94	36.8 ± 6.73	101.28 ± 18.88

Appendix 2. Data used to calculate organic carbon accumulation rates. Values were derived from previously published work and included vertical accretion from marker horizons, sediment pins and lead 210 dating, and surface elevation change from surface elevation tables (SETs).

State	Location	Mean vertical accretion (mm yr^{-1})	Surface elevation (mm yr^{-1})	Source
New South Wales	Kooragang Island	2.03 ± 0.38	192 ± 0.98	Rogers *et al.* (2013)
New South Wales	Kooragang Island	0.98 -	-	Howe *et al*. (2009)
New South Wales	Cararma Inlet	1.27 ± 0.13	3.25 ± 0.71	Rogers *et al.* (2006)
New South Wales	Minnamurra River	5.93 ± 1.21	0.26 ± 0.87	Rogers *et al.* (2006)
New South Wales	Ukerebagh	0.5 ± 0.23	0.49 ± 0.68	Rogers *et al.* (2006)
New South Wales	Berowra Creek	5.05 ± 0.72	-	Rogers (2004)
New South Wales	Homebush Bay	2.2 ± 0.29	2.92 ± 1.59	Rogers *et al.* (2006)
New South Wales	Minnamurra Creek	1.79 ± 0.56	-	Rogers *et al.* (2006)
New South Wales	Currambene	0.33 ± 0.11	0.14 ± 1.48	Rogers *et al.* (2006)
New South Wales	Port Stephens (M)	0.54 - (Pb 210)	-	Unpublished
New South Wales	Port Stephens (F)	1.75 - (Pb 210)	-	Unpublished
New South Wales	Wapengo Lake (M)	2.01 - (Pb 210)	-	Unpublished
New South Wales	Wapengo Lake (F)	0.69 - (Pb 210)	-	Unpublished
Queensland	Amity North	-	-0.08 ± 0.61	Lovelock *et al*. (2013)
Queensland	Amity South	-	0.18 ± 0.61	Lovelock *et al*. (2013)
Queensland	Adams Beach	-	5.92 ± 0.35	Lovelock *et al*. (2013)
Queensland	Tinchi Tamba Reserve	-	0.45 ± 0.32	Lovelock *et al*. (2013)
Queensland	Nundah Creek	-	1.22 ± 0.92	Lovelock *et al*. (2013)
Queensland	Halloran Reserve	-	0.11 ± 0.09	Lovelock *et al*. (2013)
Victoria	French Island	4.07 ± 0.025	5.27 ± 0.96	Rogers *et al.* (2006)
Victoria	Koo Wee Rup	2.03 ± 0.32	-0.16 ± 0.94	Rogers *et al.* (2006)
		1.63 ± 0.16 (Pb 210)	-	Rogers *et al.* (2005)
Victoria	Quail Island	2.35 ± 0.96	-0.68 ± 1.18	Rogers *et al.* (2006)
		1.4 ± 0.2 (Pb 210)	-	Rogers *et al.* (2005)
Victoria	Rhyll	1.59 ± 0.19	0.64 ± 0.75	Rogers *et al.* (2006)
		2.5 ± 0.1 (Pb 210)	-	Rogers *et al.* (2005)
Average		2.09 ± 0.32	1.44 ± 0.44	
